# Primary testicular diffuse large B-cell lymphoma: Case series

**DOI:** 10.1097/MD.0000000000019463

**Published:** 2020-03-20

**Authors:** Qi Wang, Dafang Zheng, Damin Chai, Shiwu Wu, Xiaolin Wang, Shaonan Chen, Linhui Wu, Ruoxue Cao, Yisheng Tao

**Affiliations:** a Department of Pathology; b Department of Pathology, Bengbu Medical College; c Department of Radiology, The First Affiliated Hospital of Bengbu Medical College; d Research center of Clinical Laboratory Science, Bengbu Medical College, Bengbu, Anhui, China.

**Keywords:** diagnosis, diffuse large B-cell lymphoma, primary testicular lymphoma, prognosis, treatment

## Abstract

**Rationale:**

Primary testicular lymphoma (PTL) is a rare type of extranodal non-Hodgkin's lymphoma (NHL). Although data of PTL in patients with diffuse large B-cell lymphoma (DLBCL) are accumulating, there are still patients respond poorly to prognosis.

**Patient concerns:**

All patients had disease of the DLBCL subtype and those patients had primary involvement of the testis. In our studies, eleven patients had stage I/II disease, and 3 patients had advanced disease with B symptoms. Four patients exhibited a MYC+, BCL2+, and BCL6- expression pattern, 4 patients had a MYC+, BCL6+, and BCL2- expression pattern, and 3 patients had a MYC+, BCL2+, and BCL6+ expression pattern. Additionally, 43% (7/16) of PT-DLBCL patients had a germinal center B-cell-like (GCB) phenotype, while the others had a non-GCB phonotype.

**Diagnoses:**

In our case, most patients presented with unilateral painless scrotal swelling and the enlargement of the testicles in the first examination. After hospitalization, all patients underwent preoperative imageological examination of the testis and epididymis and postoperative revealed that all patients were the diffuse infiltration of a large number of anomalous lymphocytes. In addition, no invasion of other sites was observed within 3 months after diagnosis.

**Interventions and outcomes:**

Underwent orchiectomy on the affected side was performed by urologists after all patients were diagnosed with PTL. Meanwhile, some patients received at least one course of chemotherapy, or received postoperative combined RT and chemotherapy. Because of it particularity, nineteen instances of lymph node region involvement were discovered in 12 patients since the operation.

**Lessons:**

PT-DLBCL has unique biological characteristics, and its treatment modalities are becoming increasingly standardized. In the future, systematic interventions need to be actively considered in the early stages of PTL.

## 1 Introduction

Primary testicular lymphoma (PTL) is a rare type of extranodal non-Hodgkin lymphoma (NHL), accounting for 1% to 2% of malignant lymphoma and 1% to 5% of primary testicular tumors.^[1]^ The majority of patients with this disease are categorized as having the diffuse large B-cell lymphoma (DLBCL) histological subtype, accounting for 80% to 90% of testicular lymphoma.^[2–6]^ Moreover, PTL is the most pervasive testicular neoplasm in men over 60 years of age.^[4,7]^ However, the rarer subtypes of this disease are clinically important and should also be recognized, such as Burkitt lymphoma and Burkitt-like lymphoma in patients with HIV infections. The clinical characteristics of DLBCL include low incidence, high aggressiveness, and a complicated therapeutic approach, which may explain why there are currently no standard treatments. Additionally, orchiectomy followed by chemotherapy is traditionally the recommended treatment for patients with PTL and can cure 60% to 70% of patients.^[8]^ However, there are still patients who respond poorly to treatment, experiencing frequent recurrence and poor prognosis; importantly, advanced PT-DLBCL can easily disseminate to other extranodal sites, such as the central nervous system (CNS), contralateral testis, pleura, and soft tissue.^[2,3,9,10]^

In this study, we aimed to examine the clinical and histological features, imaging presentations, IHC results, treatments, and prognostic factors in patients with PT-DLBCL and to evaluate the association of these findings with patient outcomes. In DLBCL, there have recently been significant advances in the understanding of the effects of BCL2, BCL6, and C-MYC alterations. Numerous prognostic factors have been tested in DLBCL, such as older age, high International Prognostic Index (IPI) scores, advanced stage, elevated lactate dehydrogenase, orchiectomy alone, and B symptoms.^[9,11,12]^ However, due to the rarity of PT-DLBCL and improvements in the molecular understanding, the “double expression” and “triple expression” of various markers have been recently reported in a few cases.^[13–15]^

## 2 Materials and methods

### 2.1 Ethics statement and tissue sample collection

This study was approved by the ethics committee of The First Affiliated Hospital of Bengbu Medical College (No. BBMCEC2018021), and all patients signed a written informed consent form. We retrospectively reviewed the clinical data from 1091 newly diagnosed DLBCL patients who were treated at the First Affiliated Hospital of the Bengbu Medical College between November 2006 and March 2019. In this series, 45 patients had primary involvement of the testis. Twenty nine patients were excluded because of inadequate follow-up data, and the final analysis included 16 patients. For those patients, complete clinical and pathological data were obtained (none of these patients received preoperative chemotherapy or radiotherapy (RT). The clinical information included the age, symptoms, medical history, laboratory examinations, and imaging examinations (such as scrotal ultrasound (US) and the computed tomography (CT) scans of the chest, abdomen, and pelvis). Bone marrow aspiration and cerebrospinal fluid examination was carried out in some patients, depending on the symptoms. Thus, the staging for PTL was performed using the Cotswolds-modified Ann Arbor criteria.^[16]^ In addition, the diagnosis of PTL is characterized by the analysis of the testicle and the subsidiary areas as the first diagnostic site and whether regional lymph node involvement was present or absent at diagnosis. No invasion of other sites was observed within 3 months after diagnosis in a clear pathological diagnosis.^[3,7,17]^ We also reevaluated all patients with the IPI according to the National Comprehensive Cancer Network (NCCN) clinical practice guidelines in 2017. All specimens from other hospitals were evaluated by the Department of Pathology at our hospital.

### 2.2 Histologic analysis

#### 2.2.1 Staining method

The formalin-fixed, paraffin-embedded (FFPE) samples from 16 PT-DLBCLs that were processed according to a classical histological technique, including fixation in 4% buffered formalin and embedment in paraffin. Sections were cut with a thickness of 4 microns. Then, parts of the sections were deparaffinized, dehydrated with xylene, and treated in graded ethanol.^[17]^ Immunohistochemistry was performed according to the manufacturers directions. DAB was used as a chromogen, and the slides were counterstained with hematoxylin and eosin, dehydrated, air-dried, and mounted. A known positive control for the antibodies were provided by the manufacturer. Correspondingly, phosphate-buffered saline (PBS) was used instead of the antibodies as a negative control. Meanwhile, remaining slice were available for histological and biological evaluation were analyzed, such as hematoxylin and eosin (HE) staining. Finally, a combination of IHC markers was used to further clarify the classification.

### 2.3 Analysis of staining results

The staining results were evaluated by 2 experienced pathologists with an independent double-blind method: the C-MYC, MUM1, BCL-6, PAX-5, and Ki-67 proteins were mainly expressed in the nucleus; the CD10, CD20, and CD79a proteins were mainly expressed in the cell membrane; and the BCL-2 and CD3 proteins were expressed in both the cytoplasm and cell membrane. All the positive staining results appeared as brown-yellow particles. We used a double-scoring method in which 10 high-power visual fields (×400) were selected randomly in each sample, and the percentage of positive cells in each high-power visual field was determined and scored. First, a score was given for the staining intensity: 0 for no staining, 1 for light-yellow staining, 2 for brownish-yellow staining, and 3 for tan staining. The percentage of positive cells was scored as follows: 0 for no cells, 1 for <10% positive cells, 2 for 11% to 50% positive cells, 3 for 51% to 75% positive cells, and 4 for >75% positive cells. The product of the staining intensity score and cell number score was used as the final score. If the score was less than or equal to 3, it was considered negative, and scores over 3 were considered positive. In addition, all patients with DLBCLs were classified into GCB and non-GCB subtypes according to the Hans algorithm.^[18–21]^

### 2.4 Follow-up

All patients were followed up by telephone or in the clinic. Overall survival (OS) was the measured outcome. OS was measured from the time of diagnosis to the time of death from any cause. Follow-up was completed by March 2019, with a median follow-up of 14 months. Among all patients, we failed to complete the follow-up for 3 patients because they could not be contacted and did not continue to see the doctor, with a loss rate of 12.5%. The date of the loss of follow-up was regarded as the date of death.

### 2.5 Statistical analyses

Data analysis was performed using the Statistical Package for the Social Sciences (SPSS), version 25.0. We conducted descriptive statistics for patients clinical features and IHC results.

## 3 Results

### 3.1 Clinical evaluation

The main clinical features of patients with PT-DLBCL are summarized in Table 1. The age of the 16 patients with PT-DLBCL ranged from 36- to 93-years-old, with a median age of 62-years-old, and 56% (9/16) of the patients were older than 60 years. The duration of the disease ranged from 7 days to 2 years, with an average disease course of 6 months. A typical clinical presentation was the painless enlargement of the testis, which was accompanied by a sense of puffing and swelling of the scrotum or hydrocele in some patients. Most patients who had available preoperative albumin and C-reactive protein data had results that were within the reference range. The number of cases with left and right disease in this group was similar, including 8 cases on the left side and 7 cases on the right side. In addition, synchronous bilateral testicular involvement at diagnosis was reported in 1 patient, and this patient was considered to have stage I disease.

**Table 1:**
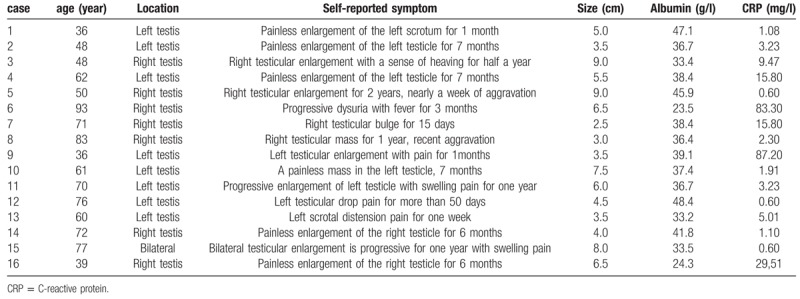
Clinical feature of 16 patients with PT-DLBCL.

### 3.2 Morphological assessment

**Figure 1:**
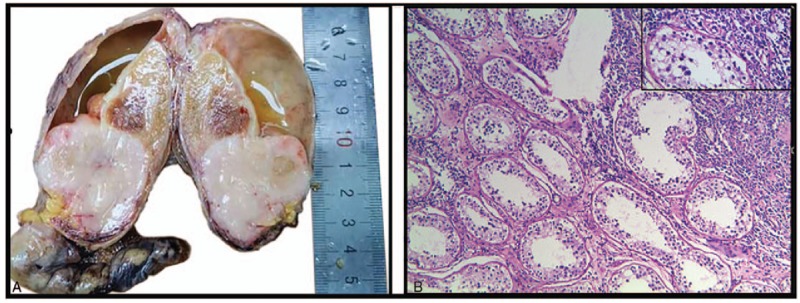
Primary diffuse large B cell lymphoma of the testis. **a**, right testicular resection specimen from a 61-year-old patient, 10 × 6 × 4.5 cm; a gray area was observed on the section, 4 × 4 × 3.5 cm **(patient 10)**. b, HE staining (original magnification, ×200; right upper quadrant, ×400) **(**patient 10**)**.

All patients underwent orchiectomy on the affected side and high ligation of the spermatic vein. Pathological examinations were performed postoperatively. In the pathological examinations, 4 cases with invasion of the epididymis and spermatic cord were identified. The macroscopic observations showed that the cut surface of the tumor was solid, and the median diameter of the testicular mass was 5.5 cm (range, 2.5–9 cm). The tumors were gray or gray-red in color, tender, and spongy and had clear boundaries (Fig. 1a). The microscopic examination showed that all the cases were DLBCL with the diffuse infiltration of a large number of anomalous lymphocytes. Fibrous hyperplasia and decreased spermatogenic components were also observed. The tumor cells were uniform and round or oval in shape and had eosinophilic cytoplasm. The nuclei were medium in size and round, oval, or irregular in shape and had 1 or more nucleoli that were visible (Fig. 1b).

**Figure 2:**
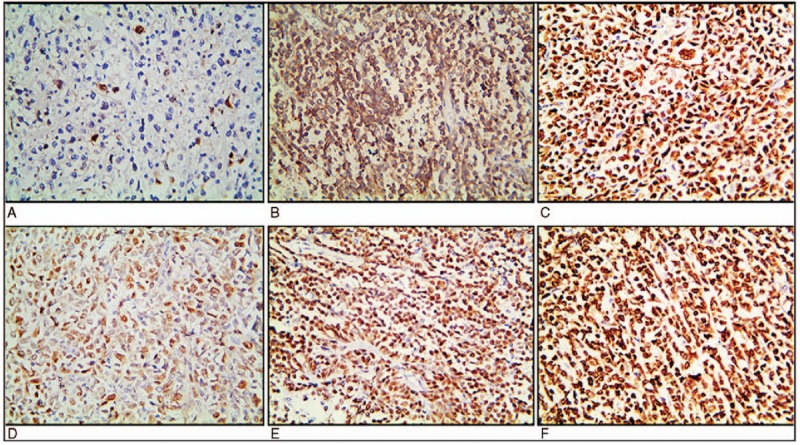
Primary diffuse large B cell lymphoma of the testis. Immunohistochemical results: a-b, CD3 and CD10 proteins were mainly expressed in the cell membrane. c-e, BCL-6, MUM1 and C-MYC proteins, respectively, were mainly expressed in the nucleus. f, BCL-2 protein was mainly expressed in the cytoplasm and cell membrane (patient 9).

We performed IHC analysis in the patients, as shown in Table 2. The IHC results of the patient tissues showed that CD20, CD79a, Pax-5, and Ki-67 were expressed, and CD3 was not expressed (Fig. 2a). In regard to the antibodies that are included in Hans algorithm, MUM1, BCL-6, and CD10 positivity were found in 9 (56%), 9 (56%), and 4 (25%) out of the 16 cases, respectively. On this basis, we classified 9 (56%) of the tumors as having a non-GCB phenotype (CD10-, BCL-6+, and MUM1+ or CD10- and BCL-6-). In addition, 7 (43%) PT-DLBCLs had a GCB phenotype, which is characteristically CD10+ (Fig. 2b); or CD10-, BCL-6+ (Fig. 2c) and MUM1 negative (Fig. 2d). In our study, the rates of C-MYC (Fig. 2e) and BCL-2 (Fig. 2f) protein expression in the 16 cases of DLBCL were eleven and ten respectively. Among these patients, 4 patients exhibited a MYC+, BCL2+ and BCL6- expression pattern, 4 patients had a MYC+, BCL6+ and BCL2- expression pattern, and 3 patients had a MYC+, BCL2+ and BCL6+ expression pattern. The mean Ki-67 index was 69% (range, 40%-90%).

**Table 2:**
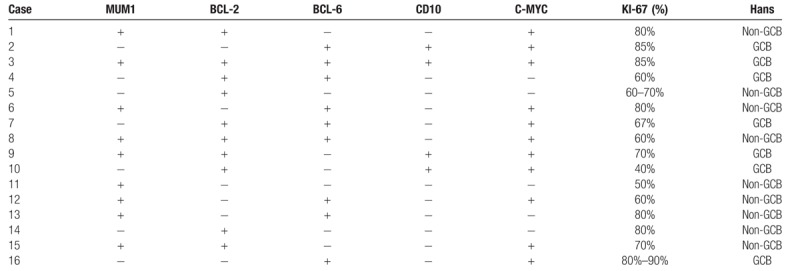
IHC results.

### 3.3 Imaging characteristics

#### 3.3.1 Ultrasonic analysis

All patients underwent preoperative ultrasonography (US) of the testis and epididymis. According to the size and distribution of the lesions, the patients were divided into the diffuse-type group and nodular-type group based on the grayscale ultrasound results.

**Figure 3:**
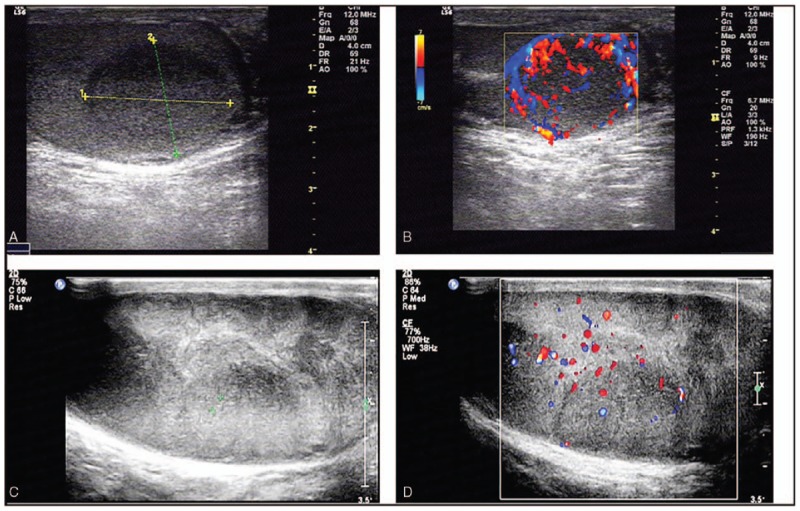
Ultrasound results: **a-b**, Limited type: The size of the right testicle was normal, 40 × 30 × 26 mm. Hypoechoic nodules with clear boundaries and regular morphology were present in the testicular parenchyma (a). CDFI showed abundant blood flow signal; Alder blood flow grade: III (patient 8) (b). c-d. Diffuse type: The volume of the left testicle was increased, 51 × 41 × 26 mm. The shape was regular, and the diffuse echo of the parenchyma was decreased. The distribution was not uniform, and there was no echo of normal testicular tissue **(c)**. CDFI showed abundant blood flow signal; Alder blood flow grade: III (patient 2) (d).

1. Six patients were placed in the nodular-type group, all of whom had unilateral onset, and the median age at the time of diagnosis was 55 years old (range, 39 to 93 years). Two patients had a single lesion in the testis, and 4 patients had 2 or more hypoechoic nodules in the upper, and lower poles of the testis. The sonographic features of nodular testicular lymphoma are a normal or slightly enlarged testis, hypoechoic nodules in the testicular parenchyma, clear boundaries, and regular morphology. Among these patients, 2 patients had relatively symmetrical internal lesions, and 4 patients had nonuniform internal echoes, with grid-like strong echoes (Fig. 3a and b).
2. A total of 10 patients were placed in the diffuse-type group; 9 of these patients had unilateral testicular involvement, and 1 patient had bilateral testicular involvement. The median age at diagnosis in the diffuse-type group was 56 years (range, 36 to 77 years). The ultrasonographic features included obvious testicular enlargement, diffuse hypoechoic involvement of almost the entire testicular parenchyma, and a linear hyperechoic lesion that could be seen in the lesion; 4 patients showed radial and parallel linear hyperechoic bands. Six patients had testicular lesions that involved the spermatic cord and epididymis. The ultrasonographic findings showed that the epididymis was obviously enlarged, the internal echo was diffuse and decreased or the strength was uneven, the normal structure of the spermatic cord was not present, and there was obvious swelling (Fig. 3c and d). Patients who underwent color dopplers showed abundant blood flow signals within the lesions; 11 testis had level III blood flow, 5 had level II blood flow.

#### 3.3.2 CT analysis

**Figure 4:**
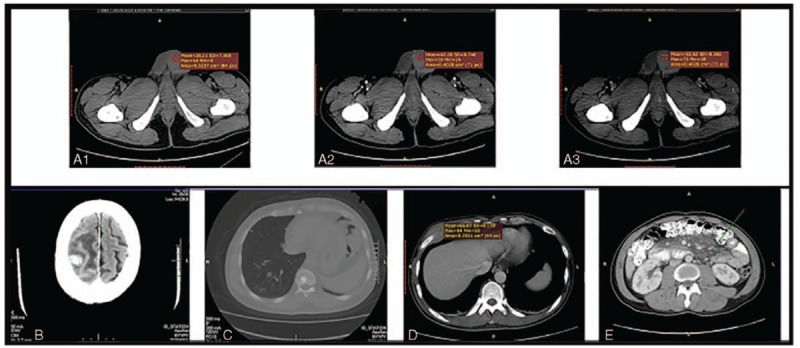
CT examination results of the testis. **a1**, A CT plain scan showed enlargement of the left testicle with a clear border and uniform density **(**patient 1**)**. **a2-3**, A CT enhanced scan showed relatively uniform density (patient 1). **b**, Right parietal lobe nodule with an abnormal density shadow, edema, and obvious enhancement **(patient 3)**. **c**, Multiple metastatic tumors in both lungs with partial vertebral metastasis **(patient 9)**. **d**, A mass with a soft tissue shadow can be observed at the right anterior rib, with a maximum diameter of 8.4 cm (patient 9). **e**, Scattered shadows of nodules can be observed in the omental bursa (patient 9).

A CT examination of the testis and scrotum was performed in 9 patients before surgery. A plain CT scan showed an enlargement of the testis on the affected side, solid space occupying the testis, and relatively symmetrical density without low-density necrosis or cystic degeneration (Fig. 4a1). A CT-enhanced scan showed that the lesion density was relatively symmetrical, and the CT value was approximately 52 to 79 HU and was increased by 10 to 30 HU. Among these patients, 6 cases of testicular lesions presented progressive enhancement in phase 3-enhanced scans, with a CT value of 45 to 95 HU in the arterial phase (Fig. 4a2) and a CT value of 58 to 110 HU in the venous phase (Fig. 4a3). Specifically, there were 3 cases of testicular lesions on the right side, 5 cases of testicular lesions on the left side and 1 case of the bilateral lesions. At the time of the initial hospitalization, 9 patients showed a thickening of the spermatic cord, tortuous and dilated internal vessels, and a small amount of testicular hydrocele in the scrotum of the affected side, including 7 cases with the invasion of the adjacent epididymis and 2 cases with tumor infiltration of the spermatic cord. Moreover, 6 patients also experienced retroperitoneal and inguinal lymph node enlargement. Unfortunately, at the first examination, 2patients had brain metastasis (Fig. 4b). One patient exhibited lymph node metastasis in the left inguinal region, lumbar vertebrae (Fig. 4c), right anterior costal region (Fig. 4d), and omental bursa, with scattered nodular shadows (Fig. 4e).

#### 3.3.3 Evaluation of treatment

The treatment and prognosis of PT-DLBCL patients are shown in Table 3. In our study, 6 (38%), 5 (31%), 4 (25), and 1 (6%) patients were diagnosed with Ann Arbor stage I, II, III, IV disease, respectively. The IPI score of 6 patients was medium-high/high risk. All patients underwent orchiectomy on the affected side as the initial therapeutic and diagnostic intervention. Because of the economic conditions and older age, 5 patients did not pursue further treatment. Eleven patients received at least 1 course of chemotherapy. Specifically, 6 patients received postoperative combined RT and chemotherapy. Among these patients, 5 patients (30%) received the CHOP protocol (cyclophosphamide 750 mg/m^2^ intravenously (IV), doxorubicin 60 mg/m^2^ IV, and vincristine 1.4 mg/m^2^ IV on day 1 and prednisone 100 mg/m^2^ orally on days 1–5 of each cycle), and 6 patients (38%) received the R-CHOP protocol (rituximab 375 mg/m^2^ IV on day zero, and cyclophosphamide, doxorubicin, vincristine, and prednisone; 3 weeks for a course of treatment, usually with a 2 week interval). Patients were assessed after 4 courses to select the next treatment. In addition, the RT dose was 25 to 30 Gy, and the RT scope was focused on the high-risk areas of the contralateral testis, groin, pelvic cavity, and posterior peritoneum. Among the patients, 6 patients (6/11) were treated with rituximab and scrotal RT after 2011, and 3 patients were treated with a prophylactic intrathecal injection (ITH). Of the 16 patients, 13 relapsed; 19 instances of lymph node region involvement were discovered, including mediastinal (3), cervical (5), inguinal-femoral (6), axillary or pectoral (2), mesenteric (1) infraclavicular (1), and lumbar lymph nodes (1). Five instances of extranodal region involvement were discovered, including bone marrow (3), brain (1) and intestine (1). Additionally, the information on the specific location of 4 patients metastases was incomplete.

**Table 3:**
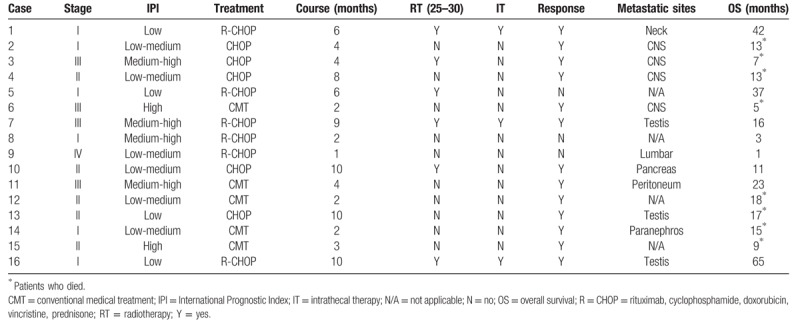
Treatment and prognosis of PT-DLBCL patients.

## 4 Discussion

PTL is a rare neoplasm with an annual incidence of 0.26 cases per 100,000 person-years.^[22,23]^ The median age of presentation of testicular lymphoma is 60 years, and the incidence is increasing. However, in recently published reports, the patients were younger than those in past cases.^[24,25]^ In our study, 5 patients were under 50-years-old, and younger patients were seen more frequently among patients who were initially seen after 2011. In the first examination, most patients presented with unilateral painless scrotal swelling and the enlargement of the testicles, sometimes with sharp scrotal pain. Systemic B symptoms (including fever, night sweats, and an unexplained weight loss of more than 10% of the body weight in 6 months) are observed in 25% to 41% of patients with advanced-stage disease.^[7]^ In our case, 3 advanced patients experienced B symptoms.

In previous studies have suggested that lymphoma can easily infiltrate the epididymis and spermatic cord. In addition, PTL has a propensity to invade other extranodal sites, such as the CNS, contralateral testicle (particularly in patients who have undergone orchiectomy alone), subcutaneous tissue, Waldeyer ring and lung.^[22,25,26]^ In our case, 13 (81%) patients relapsed: 19 instances of lymph node region involvement were discovered, including mediastinal (3), cervical (5), inguinal-femoral (6), axillary or pectoral (2), mesenteric (1) infraclavicular (1) and lumbar lymph nodes (1). Five instances of extranodal region involvement were discovered, including bone marrow (3), brain (1) and intestine (1). The findings of these cases appear to be consistent with retrospective reports. However, the small samples size in this study will lead to insufficient statistical efficiency. Meanwhile, the involvement of these sites may occur simultaneously or later in the course of the disease. These types of involvement are believed to be associated with the common embryonic origin^[7]^; changes in the tumor microenvironment (TME)^[27,28]^; limited immune surveillance during the pathogenesis of lymphoma^[29]^; and gene translocations or anatomic proximity,^[26]^ especially in patients with disease of non-germinal center B cells.^[25,30]^

In larger retrospective reports, several other prognostic factors for testicular lymphoma have been described. In 2003, the International Extranodal Lymphoma Study Group (IELSG) found that older age, advanced stage, high IPI score, elevated lactate dehydrogenase, and not having surgery or RT were significantly associated with poor prognosis.^[3]^ In 2009, Gundrum and his colleagues showed that older age, advanced stage, and left testicular involvement were associated with a worse prognosis.^[11]^ In 2010, researchers at MD Anderson Cancer Center concluded that advanced stage, elevated lactate dehydrogenase, and high IPI were predictors of disease survival.^[12]^ In 2011, Battista and his partners found that first-line treatment with R-CHOP, IT-MTX, and testicular RT was associated with a better outcome.^[31]^ In 2012, another study that included 167 cases evaluated the expression of MYC and BCL-2 and showed that high-grade B cell lymphoma with concurrent MYC, BCL-2 and BCL-6 rearrangements (“triple-hit”) and high IPI had poor prognosis.^[14]^ In 2013, a retrospective study that included 893 cases concluded that CNS-IPI and non-GCB had a worse prognosis than that of the germinal center B-cell phenotype.^[32]^ In 2018, Thomas A and his colleagues found that B symptoms, intravascular lymphoma, and concurrent MYC, BCL-2 or/and BCL-6 rearrangements (“double-hit” or “triple-hit”) are often associated with shorter survival.^[26,33]^ Several studies also have investigated the effect of the specific expression patterns of BCL2, BCL6 and MYC in PT-DLBCL, which have been shown to be associated with poor patient outcomes.^[13,14,34,35]^ In 2019, a population-based largest retrospective study in China confirmed that age, year of diagnosis, Ann Arbor stage, phenotype, RT and rituximab may provide survival benefits.^[5]^ In our study, 11 (69%) patients were diagnosed with testicular lymphoma at early stage. And the IPI score of 6 patients was medium-high/high risk. Because of the economic conditions and older age, 5 patients did not pursue further treatment. Surprisingly, as the medical environment improved, patients with insurance or with Medicaid had better survival rates than those without; especially, patients who were treated after 2011. Meanwhile, several published studies suggested that insurance status were inversely associated with survival in patients with several cancers.^[36–40]^ Because most cases of PT-DLBCL present with stage I/II disease.^[3,26]^ By definition, it is difficult to distinguish stage III/IV PTL and systemic DLBCL with secondary testicular involvement. Therefore, image-assisted examination plays an important role in diagnosis.

US is the most common imaging method used for testicular masses. PTL presents focal or diffuse hypoechogenicity with vascular proliferation. In our study, 6 patients were placed in the nodular-type group, all of whom had unilateral onset; ten patients were placed in the diffuse-type group, 9 of these patients had unilateral testicular involvement and 1 patient had bilateral testicular involvement. Among the testis lesions within the blood vessels, the go line was disordered, and the characteristics of more nourishing blood vessels were observed inside the malignant testicular lymphoma. For patients with ambiguous ultrasound results, a pathological examination combined with IHC analysis is preferable. In recent years, CT or magnetic resonance imaging (MRI) has also played an important role in the diagnosis of primary testicular tumors by simultaneously evaluating the structure of the testis and epididymis.^[9,41]^ At the same time, there is growing interest in using fluorescence in situ hybridization (FISH) as the gold standard for diagnosis^[24]^; however, this method is rarely employed because of patient preference and economic conditions in our case.

Historically, orchiectomy has been the method of choice for improving prognosis. Orchiectomy not only provides histological samples for diagnosis but also removes the neoplastic mass. Above all, orchiectomy removes the potential sanctuary site. In the presence of the blood-testicular barrier, it is difficult for drugs to enter the testicles, and the effect of chemotherapy is not ideal^[29]^; at the same time, testicular tumor cells can also express high levels of drug-resistant proteins, such as P-glycoprotein (P-GP) and breast cancer drug-resistant protein (BCRP), which results in chemotherapy resistance.^[42]^ Although orchiectomy alone can sometimes lead to long-term survival, orchiectomy should not be used as the only surgical treatment, even in stage I patients. In relevant reports, most patients who received surgical treatment relapsed within 2 years.^[3,7]^ In our study, 3 patients died at 9, 15, and 18 months after surgery, respectively. Thus, orchiectomy followed by chemotherapy (CHOP or CHOP-like regimens), local RT and preventive CNS intrathecal injection are widely accepted options, which are suitable for individual clinical use with type-3 levels of evidence.^[26]^ The use of multimodal therapy was associated with an increase in the 5-year survival rate from 30 to 86.6%.^[3,5,7,23,43]^ In our study, the OS of patients who were treated after 2011 was significantly improved compared with that of patients who were treated between 2006 and 2010. In particular, patients with stage I/II disease were treated with more than 4 courses of CHOP and rituximab. Fortunately, the significance of the expression of IHC biomarkers, such as the Bcl-2, bcl-6, and MYC proteins, in tumor cells has also attracted much attention. In the future, molecular targeted therapy is expected to be applied in the clinic to improve the prognosis of patients, such as chimeric antigen receptor T-cell therapy, PD-1and PD-L1 pathway.^[28,44–47]^ Our research group will study the mechanism of action of the BCL-2, BCL-6, and MYC proteins at the cellular level.

Our retrospective study has several limitations. First, because some data were missing, it was difficult to analyze some of reported prognostic predictors. Second, the results described above need to be confirmed by larger datasets because of the small study population. Third, because of the relatively short follow-up period, it was difficult to identify the best dose and regimen for chemotherapy. Finally, because our samples were collected over 13 years, the treatment methods changed several times, and the OS was worse than that in other retrospective studies. The worsened OS could be explained by a lack of economic support, an older age, and the shame that is associated with consulting a doctor for a genital-related disease. However, considering the unique nature of PT-DLBCL, this review still provides useful information for clinical diagnosis.

## 5 Conclusion

In conclusion, PT-DLBCL has unique biological characteristics (low incidence, high aggressiveness, and complicated therapeutic approach). Fortunately, its treatment modalities are becoming increasingly standardized. Future use of molecular-targeted therapy in clinical practice and prospective studies are expected to improve the prognosis of patients. Moreover, systematic interventions need to be actively considered in the early stages of PTL.

## Acknowledgments

We thank Professors Ze-nong Cheng and Cong-you Gu at the Department of Pathology (Bengbu Medical College) for their kind help in the determination of the IHC profile of primary testicular lymphoma. Furthermore, we are also grateful to the statistics teacher who did the data analysis.

## Author contributions

**Conceptualization:** Yi-sheng Tao.

**Data collection:** Qi Wang.

**Formal analysis:** Qi Wang, Da-fang Zheng.

**Funding acquisition:** Yi-sheng Tao, Da-min Chai

**Investigation:** Qi Wang.

**Project administration:** Xiao-lin Wang, Ruo-xue Cao, Lin-hui Wu.

**Imaging data:** Shao-nan Chen.

**Supervision:** Shi-wu Wu.

**Validation:** Shi-wu Wu.

**Writing – original draft:** Qi Wang.

**Writing – review and editing:** Qi Wang.

Qi Wang orcid: 0000-0001-8786-292X.
